# Utilization of *Biang* Fish Flour (*Ilisha elongata*) as an Enrichment Material for Sago Noodles Nutrient Value

**DOI:** 10.1155/2022/8746296

**Published:** 2022-09-10

**Authors:** Suparmi Suparmi, Sumarto Sumarto, Umi Afriana, Taufik Hidayat

**Affiliations:** ^1^Department of Aquatic Product Technology Faculty Fisheries and Marine Science, Riau University, Pekanbaru, Indonesia; ^2^Research Center of Agroindustry, National Research and Innovation Agency, Jakarta, Indonesia

## Abstract

This study aims to determine the appropriate concentration of lead fish meal for enriching the nutritional value of sago noodles favored by consumers. The method used is an experimental design using Completely Randomized (CRD) with 4 concentration levels of lead fish meal, namely, 4% without a lead fish meal (M0), 6% (M1), 8% (M2), and 10% (M3). The analysis of sago noodles was performed and proximate by the AOAC method; amino acids by HPLC; fatty acids by GC; and minerals by HPLC. The results of the showed that the study getting the best treatment was a concentration of 8% (M2) with the characteristics of whole sago noodles' appearance: attractive, grayish-white color; a distinctive aroma of sago noodles with a hint of fish; a specific taste typical of sago noodles and fish prickly taste; delicious; slightly chewy texture. Sensory evaluation with a taste value of 8.9, an aroma of 8.6, a visual value of 8.9, and a texture value of 8.8. Its nutritional content is 5.58% protein content, air 22.35%, ash 1.69%, fat 1.41%, and carbohydrates 68.29%. The proximate values are protein 5.58%, water content 22.35%, ash 1.69%, fat content 1.41%, and carbohydrates (different) 68.29%. The mineral content is Ca.P.I, Mg, Zn, and Fe. Amino acids consist of 8 types of essential amino acids, namely, histidine, arginine, threonine, valine, alanine, methionine, isoleucine, leucine, phenylalanine, and 7 types of nonessential amino acids, namely, aspartic acid, glutamic acid, serine, glycine, tyrosine. Its fatty acid profile has 13 components of unsaturated fatty acids and 17 components of saturated fatty acids.

## 1. Introduction

Sago noodles are a special food and are in demand by the community, especially in Kab, Meranti Islands, Riau Province, Indonesia, because noodles are easy to serve, durable, and relatively cheap [1]. Indonesian people have experienced changes in consumption patterns, namely, by making noodles as a companion or substitute for rice. Sago noodles are made from sago flour, which contains 84.7% high carbohydrate content and 353 kcal energy, but has a low protein content of 0.7%, 0.2% fat content, and low mineral content. However, sago flour as the basic ingredient for making noodles has several advantages compared to other flours; namely, it contains undigested starch and is beneficial for digestive health; namely, “resistant starch” (RS), which has effects such as dietary fiber and the use of fishery products in the form of fortification can increase the added value of the product [2].

Processed sago noodles can be produced with applicable technology and several raw materials, for example, surimi powder and additional fish meal [3,4]. Efforts to increase the nutritional value of sago noodles are by fortifying highly nutritious ingredients such as fish meal, which has a high nutritional content where the protein content reaches 73.0%, water content 7.12–7.88%, ash content 12.97 > 11.89%, fat content 8%, and has minerals that are important for the growth and development of the body. Among others, the fish meal contains calcium (Ca) 8397–8402 mg/kg, phosphorus (P) 169–183 mg/kg, iodine (I) 189.44–190.16 mg/kg, magnesium (Mg) 141.32–141.88 mg/kg, zinc (Zn) 152.07–153.02 mg/kg, and iron (Fe) 15.76–15.93 mg/kg; therefore this lead fish meal has the potential to be developed, as well as being able to act as a fortification of other food products based on the fish meal as an effort to increase the nutrition of protein-rich foods [5]. Fish meal from biang fish, which is an endemic fish in Riau Province contains high protein.

The addition of processed fish-based products is one of the efforts to increase fish consumption in the community. Processed fish products developed must lead to products that can be eaten immediately (ready to eat), are easy to carry, and do not take long to cook. Therefore, this study aimed to determine the concentration of the main flour in increasing the nutritional value of sago noodles.

## 2. Materials and Method

### 2.1. Material and Tools

The main ingredients used in this study were 50 kg of fresh pike fish weighing 77–95 g, water, and sago flour. The chemicals used for proximate analysis were methanol, sodium acetate, triethylamine, sulfuric acid, equates, eluent, sodium acetate buffer, boric acid, sodium hydroxide, hydrochloric acid, PP indicator, diethyl ether, catalyst, and blue reagent.

The tools used in this research are oven, grinder, blender, jar, tray, 80 mesh sieve, basin, spoon, knife, presto, analytical scale, sago noodle ampia machine, sago noodle printing machine, frying pan, pan, basin, dough machine, sago noodles, plastic packaging, label paper, gas stove, stove, and press.

### 2.2. Research Methods

The method used in this research is an experimental design. The research method includes manufacturing of lead fish meals and making sago noodles fortified fish flour.

#### 2.2.1. Manufacture of Lead Fish Meal (Modification [5])

The whole gizzard was removed and washed thoroughly, then pressed for 60 minutes, then dried using an oven for 48 hours at 44.20 C, and made flour using a blender. To obtain a uniform flour grain size, filtering is carried out using an 80-mesh sieve.

#### 2.2.2. Making Sago Noodles Fortified Fish Flour (Modification [5])

The gelatinization process of sago flour is done by adding a little water to the sago flour (the process of moisturizing the material) until smooth, then the flour is roasted for about 5–10 minutes. The process of mixing noodle dough ingredients (formulation of ingredients): sago flour 500 g, water 150 mL, a fish meal with treatment level for the M0 treatment, namely, without a fish meal, M1 fish meal 6%, M2 fish meal 8%, and M3 flour fish starter 10%, stirred until perfect gelatinization is formed. Then the dough is in the form of small balls and placed in plastic to give a boundary. Printing of dough: the dough is formed into small circles and delimited and then printed using amphibia to form a sheet.

The results of the dough mold are wrapped in long plastic sheets and then a short boiling process is carried out by inserting the dough mold in a container filled with boiling water until cooked, marked with a brownish yellow color (1–2 minutes). The process of draining and aerating in a closed room for 12 minutes. o'clock. The formulation of the fortified sago noodles of the prickly fish flour can be seen in Table 1.

### 2.3. Procedure Analysis

#### 2.3.1. Organoleptic Test (see[6])

The organoleptic test was carried out by 25 moderately trained panelists to test the quality of sago noodles fortified with lead fish meal. The organoleptic test usually aims to determine the panelists' responses to the general quality properties of color, aroma, texture, and taste by using a score sheet on a scale of 1 as the lowest value and 9 as the highest value.

#### 2.3.2. Proximate Analysis (see[7])1

The proximate analysis includes moisture, ash, protein, fat, and carbohydrate content.

*(1) Analysis of Calcium Levels* [8]. Calcium level testing: the determination of calcium levels was carried out by measuring the wet-digested sample using an Atomic Absorption Spectrophotometer (AAS) at a wavelength of 420 nm. The calcium content was tested by referring to the modified method. Analysis of the calcium content of the sample was carried out by weighing 0.1 g of the fine sample and transferring it to a 100 mL volume Kjeldahl flask. Sample destruction was carried out by fortifying 15 mL of hydrochloric acid (HCl). The solution was digested until it became clear and then cooled. The filtered volume is calibrated to 100 mL and ready to be measured on AAS.

*(2) Analysis of phosphorus levels* [8]. Phosphorus content was detected using a U-VIS spectrophotometer, in which the test method referred to was modified. The sample was weighed as much as 5 g, added 20 mL of concentrated HNO_3_, then boiled for 5 minutes, and cooled, then added 5 mL of concentrated sulfuric acid (H_2_SO_4_). The solution was heated and refined (digestion) with HNO_3_ fortification drop by drop until the solution was colorless, followed by heating until white smoke appeared and cooled. Add 15 mL of distilled water to the beaker and boil again for 10 minutes. A total of 10 ml of the sample solution was put into a 100 mL volumetric flask. Then 40 mL of distilled water and 25 mL of vanadate molybdate reagent were added to the measuring flask and diluted to the mark. The absorbance value of the solution was measured by a spectrophotometer at a wavelength of 400 nm.

### 2.4. Analysis of Data

The experimental design used was a nonfactorial Completely Randomized Design (CRD) with 4 levels, namely, M0 (without fish meal), M1 (6% fish meal), M2 (8% fish meal), and M3 (10% fish meal) Then, each treatment was repeated 3 times. Organoleptic test with Kruskal–Wallis test with 30 untrained panelists.

## 3. Result and Discussion

### 3.1. Characteristics of Mi Sago Fortified with Fish Flour

The results of the assessment of the characteristics of sago noodles fortified with a fish meal from organoleptic parameters, proximate, mineral content, and amino acid content can be seen in the following description.

#### 3.1.1. Sensory Evaluation

The results of the organoleptic test analysis of the fortified sago noodles of starter fish meal can be seen in Table 2.

**Figure 1:**
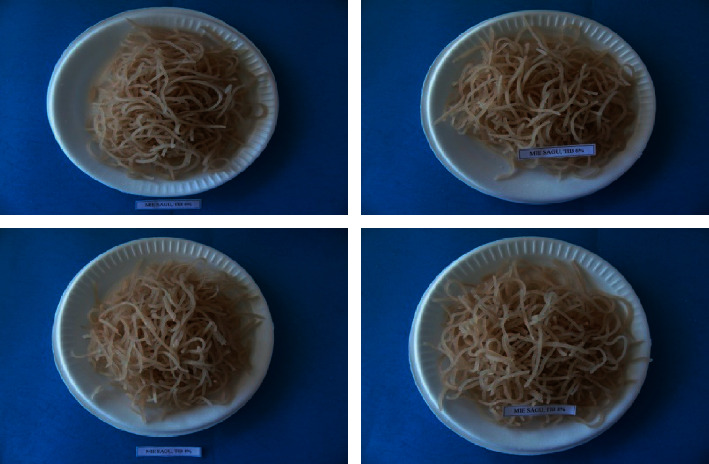
Photograph sago noodles (M_o_, M_1_, M_2_, and M_3_).

In Table 3, the average value of the appearance of sago noodles with the fortification of starter fish meal ranged from 5.03–8.19. The highest average appearance value was found in sago noodles without fortification of lead fish meal M2 (8.19) with intact, attractive characteristics, bright white color, and the lowest M0 (5.03) with characteristics of being easily broken, cracked, less attractive, and dull white. M1 (5.99) with intact characteristics, somewhat attractive, slightly bright white color, and M3 (6.96) with intact characteristics, easy to break, slightly attractive, slightly bright white color (Figure 1).

The results showed that there was an effect of fortification of the lead fish meal on the organoleptic quality characteristics (appearance, smell, taste, and texture) of sago noodles. Sago noodles fortified with 8% fish meal had the best appearance, taste, aroma, and texture values from all treatments. This is the opinion of [9], which states that the limit on the amount of fortification of scad fish meal if the amount is >8% causes the low organoleptic value of noodles. According to [10], the higher the fortification of fish meal on sago noodles will produce an appearance that makes the sago noodles pale and not bright so that the visual value of fish meal fortified sago noodles shows differences, where the appearance of each treatment will become paler and not bright.

The phenomenon of change in organoleptic value is caused by a nonenzymatic reaction (the Maillard reaction). Maillard reactions can be triggered by heating at high temperatures, such as roasting, frying, roasting, and cooking processes [11].

#### 3.1.2. Proximate

The data from the proximate analysis of sago noodles fortified with a fish meal can be seen in Table 3.

Table 4 showed that the average protein content of sago noodles fortified with the prickly fish meal with different concentrations ranged from 0.45–7.49%. The highest average protein content was found in sago noodles with 10% lead fish meal fortification (M3), which was 7.49%, and the lowest protein was found in treatment (M0), which was 0.45%. The results of the analysis of variance showed that the fortification of the lead fish meal had a significant effect on the protein content of sago noodles, where F count (610.42) > F table (4.07) at the 95% confidence level. M0 (0.45%) was significantly different from the treatment M1 (5.58%), M2 (6.34%), and M3 (7.49).

The results showed that the fortification of lead fish meal in sago noodles had a significant effect on the average value of protein content in sago noodles. The higher the amount of fish meal fortified in sago noodles, the higher the average protein content, because the protein content of the fish meal reaches 73.0% [5]. Furthermore, [12] stated that fish contains high protein and is composed of amino acids that the body needs for growth. Fish protein is very easy to digest and absorb by the body. The protein content of sago noodles fortified with Biang fish meal in the M1, M2, and M3 treatments met the SNI requirements of 3% [13].

Based on the results of the water content test in Table 4, shows that the average value of the water content of sago noodles fortified with different concentrations of fish meal is between 21.04 and 22.65%. The highest water content was found in sago noodles without fortification of 0% fish meal (M0), which was 22.65%, and the lowest was found in sago noodles with the fortification of 10% fish meal (M3), which was 21.04%. The results of the analysis of variance showed that the fortification of the lead fish meal had a significant effect on the water content of sago noodles, where F count (5.59) > F table (4.07) at the 95% confidence level. The results of the further BNJ test showed that each treatment had a significantly different water content. The results of this study indicate that the greater the concentration of fortified starter fish meal in sago noodles, the lower the water content of sago noodles. According to [14], this is due to the hygroscopic fortification of fish meal, which is a binder used by the food industry to bind/absorb the water content in the dough. However, the average water content value of sago noodles is still acceptable because, based on the maximum standard, it is still in accordance with the standard SNI 01-2987-1992, the moisture content of semi wet noodles is 20–35%.

Based on Table 3, shows that the average fat content of fortified sago noodles with different concentrations of starter fish meal is between 0.36 and 1.69%. The highest average fat content was found in sago noodles with a 10% (M3) fortification of starter fish meal. Meanwhile, the lowest average fat content was found in sago noodles without fortification of M0 starter fish meal (0%).

The results of the analysis of variance showed that the fortification of the lead fish meal had a significant effect on the fat content of sago noodles, where F count (87.94) > F table (4.07) at a 95% confidence level. A further BNJ test found that the treatment M0 (0.33%) was significantly different from the treatments of M1 (1.45%), M2 (1.59%), and M3 (1.69%). M1 (1.45) was not significantly different from M2 (1.59%), and M2 (1.59%) was also not significantly different from M3 (1.69%).

The results showed that the fortification of the root fish meal in sago noodles had a significant effect on the average value of fat content in sago noodles. The higher the amount of fish meal fortified in sago noodles, the higher the average value of the fat content because the fat content of the fish meal is between 5.96 and 6.69% [15]. Fat content is very influential on the durability of the material; if the fat content of the material is high, it will accelerate rancidity due to fat oxidation [16]. The fat content of sago noodles fortified with a fish meal in all treatments met the SNI requirements of <7% [13].

Based on Table 3, shows that the average value of the ash content of sago noodles fortified with different concentrations of fish meal is between 0.72 and 2.43%. The highest average ash content was found in sago noodles with the fortification of lead fish meal 10% (M3), which was 2.43%. Meanwhile, the lowest average ash content was found in sago noodles without fortification of lead fish meal (M0), which was 0.72%.

The results of the analysis of variance showed that the fortification of the lead fish meal had a significant effect on the ash content of sago noodles, where F count (290.89) > F table (4.07) at a 95% confidence level. The results of the BNJ follow-up test showed that the treatment of M0 (0.72%) was significantly different from the treatments of M1 (1.69%), M2 (2.15%), and M3 (2.43%). While the treatment of M2 (2.15%) was not significantly different from P3 (2.43%), the results showed that there was an effect of the fortification of lead fish meal on the average value of ash content in sago noodles. The higher the amount of fortified fish meal, the higher the average ash content. This is because the mineral content in the lead fish meal reaches 2.94 bb% [17]. According to [18], most foodstuffs (96%) consist of organic and water. In the process of processing (burning), organic matter burns but inorganic substances do not; therefore higher levels of mineral elements are detected. The results showed that the ash content produced was in accordance with the quality standard of semi wet noodles, namely a maximum of 3% [19].

Based on Table 3, shows that the average value of the carbohydrate content of sago noodles fortified with different concentrations of fish meal is between 67.35 and 75.85%. The highest average carbohydrate content was found in sago noodles with the fortification of 0% lead fish meal (M0), which was 75.85%, while the lowest average was (M3), which is 67.35%. The results of the analysis of variance showed that the fortification of the lead fish meal had a significant effect on the carbohydrate value of sago noodles, where F count (202267.53) > F table (4.07) at a 95% confidence level.

From the results of the study, it was known that the fortification of the root fish meal in sago noodles had a significant effect on the average value of carbohydrate content in sago noodles. The higher the amount of fermented fish meal fortified in sago noodles, the lower the average value of the carbohydrate content due to the reduced product constituent components, which are a source of carbohydrates. Carbohydrates that are high in sago starch [20]. The carbohydrate content of fortified sago noodles from the starter fish meal in all treatments met the SNI requirements, which was 86.9%.

Based on the characteristics of the organoleptic and proximate parameters, it can be seen that the M2 treatment, namely the formulation of 8% fortification of lead fish meal in sago noodles, was a treatment that met the standards and continued to analyze the parameters of mineral content, fatty acids, and amino acids. The results of the M2 treatment parameters are also compared with the control results.

#### 3.1.3. Mineral Content of Fortified Sago Noodles Starfish Flour 8%

Based on the mineral analysis that has been carried out on sago noodles with the fortification of 8% lead fish meal, the average results of the mineral analysis are shown in Table 4.

Based on Table 4, shows that the mineral is Ca.P. I, Mg, Zn, and Fe sago noodles fortified with the lead fish meal with a concentration of 8%. The results showed that the fortification of lead fish meal in sago noodles had a significant effect on the average value of calcium levels in sago noodles. The higher the amount of fish meal fortified in sago noodles, the higher the average calcium content, because the calcium content of the fish meal reaches 8397 mg/kg [21].

#### 3.1.4. Amino Acid Content of Fortified Sago Noodles Starfish Flour 8%

The results of the amino acid analysis of sago noodles fortified with 8% Biang fish meal can be seen in Table 5.

In Table 5, it can be seen that the types of amino acids for fortified sago noodles from fish meal consist of 8 types of essential amino acids, namely histidine, arginine, threonine, valine, alanine, methionine, isoleucine, leucine, and phenylalanine. There are 7 types of nonessential amino acids, namely aspartic acid, glutamic acid, serine, glycine, and tyrosine. In accordance with the results of [14] research, most of these amino acids were detected in fishery commodities. Based on the amount of amino acid content, 8% fortified sago noodles can be classified as nutritious food because they contain complete amino acids.

According to [22], each essential amino acid has a special function, namely as a cell-forming, and can also be useful as a flavor giver. In line with the opinion of [23], that the use of amino acids can be seen from the characteristics of the taste, some amino acids have a sweet taste, some a bitter taste, and some have no taste. Glycine, proline, alanine, hydroxyproline, valine, and serine have a sweet taste. Isoleucine and arginine have a bitter taste, a savory taste caused by glutamic acid, and leucine is tasteless.

#### 3.1.5. Fatty Acid Content of Fortified Sago Noodles Starfish Flour 8%

Table 6 shows that the fatty acid profile in fortified sago noodles from 8% fish meal shows a balanced portion of unsaturated and saturated fatty acids. The content of fatty acids in 8% fortified sago noodles from prickly fish flour has 13 components of unsaturated fatty acids, while saturated fatty acids have 17 components. The results of the analysis show that the fatty acid profile shows a balanced portion between unsaturated fatty acids and saturated fatty acids.

The combination of sago and biang fish flour of sago flour causes an increase in the fatty acid content of sago noodles. The highest content is found in palmitic acid. Palmitic acid can be a precursor to omega-3, which is very beneficial for human health. An increase also occurred in unsaturated fatty acids, especially for the fatty acids EPA and DHA. The increase in the fatty acid value is thought to be due to the fat content of the lead fish, which reached 19%, plus the fat content of sago, which contributed 0.2%. Sago noodles with the addition of starter fish meals can potentially be a functional food for stunting prevention [24].

## 4. Conclusion

Based on the results of the study, it can be concluded that the fortified sago noodles of sago fish meal had a significant effect on the organoleptic characteristics and its proximate content. The best treatment was fortified sago fish meal 8%, a Hedonic test with a taste value of 8.9, an aroma of 8.6, the visual value of 8.9, and a texture value of 8.8. The mineral content is Ca.P.I, Mg, Zn, and Fe. The essential amino acid's high content was methionine, and the nonessential amino acid's high content was glutamate acid. Besides that, sago noodles contain high content of EPA and DHA, which are good for health.

## Figures and Tables

**Table 1:** The formula for making sago noodles.

Material	Unit	Treatment			
		M_0_	M_1_	M_2_	M_3_
Sago flour	G	500	500	500	500
*Biang* fish flour	% (b/b)	0	6	8	10
Water	mL	150	150	150	150

**Table 2:** The average organoleptic value of fortified sago noodles from fish meal.

Treatment	Parameters			
	Appearances	Odor	Taste	Texture
M_0_	5.03^d^	5.16^c^	5.83^a^	5.24^c^
M_1_	5.99^c^	5.99^b^	7.93^c^	5.99^b^
M_2_	8.19^b^	8.45^b^	8.48^c^	8.35^b^
M_3_	6.96^a^	5.09^a^	6.67^b^	6.61^a^

Description: M0 (control), M1 (fish meal 6%), M2 (fish meal 8%), and M3 (fish flour 10%). Mean values; the different letters within the same column represent significantly different values (*p* < 0.05).

**Table 3:** The results of proximate analysis of sago noodles fortified with Biang fish meal.

Proximate	M_0_ (0%)	M_1_ (6%)	M_2_ (8%)	M_3_ (10%)
Moisture	22.65 ± 0.1^a^	22.35 ± 0.4^a^	21.72 ± 0.3^b^	21.04 ± 0.15^b^
Ash	0.72 ± 0.3^a^	1.69 ± 0.21^b^	2.15 ± 0.4^bc^	2.43 ± 0.25^c^
Protein	0.45 ± 0.4^a^	5.58 ± 0.2^b^	6.34 ± 0.26^c^	7.49 ± 0.34^d^
Fat	0.33 ± 0.2^a^	1.45 ± 0.22^b^	1.5 ± 0.13^bc^	1.69 ± 0.13^c^
Carbohydrate	75.85 ± 0.3^a^	68.93 ± 0.4^b^	68.29 ± 0.21^b^	67.35 ± 0.23^c^

Description: M0 (control), M1 (fish meal 6%), M2 (fish meal 8%), M3 (fish flour 10%). The different letters within the same column represent significantly different values (*p* < 0.05).

**Table 4:** The mineral content of 8% fortified sago noodles in fish flour.

Minerals	Mo(%)	M_2_(%)
Calcium (mg/kg)	1131 ± 0.8^a^	8402 ± 0.65^b^
Phosphor (%)	111.14 ± 0.2^a^	183 ± 0.34^b^
Iodium (I)	118 ± 0.5^a^	190.16 ± 0.56^b^
Magnesium (mg/kg)	20.4 ± 0.7^a^	141.88 ± 0.4^b^
Zinc (Zn) (mg/kg)	102.6 ± 0.13^a^	153.02 ± 0.24^b^
Iron (Fe) (mg/kg)	10.4 ± 0.25^a^	15.93 ± 0.21^b^

Description: M0 (control), M2(fish meal 8%): the different letters within the same column represent significantly different values (*p* < 0.05).

**Table 5:** Amino acid profile of sago noodles fortification of prickly fish meal.

Amino acids	M_0_(%)	M_2_(%)
1 aspartic acid	0.15 ± 0.1^a^	0.50 ± 0.3^b^
1. Threonine^*∗*^	0.07 ± 0.3^a^	0.24 ± 0.1^b^
2. Serine	0.06 ± 0.4^a^	0.20 ± 0.2^b^
3. Glutamate	0.00 ± 0.6^a^	0.88 ± 0.13^b^
4. Glycine	0.08 ± 0.3^a^	0.26 ± 0.2^b^
5. Alanine	0.10 ± 0.2^a^	0.32 ± 0.3^b^
6. Valine^*∗*^	0.10 ± 0.4^a^	0.32 ± 0.5^b^
7. Methionine^*∗*^	0.24 ± 0.1^a^	0.72 ± 0.56^b^
8. Eleusine^*∗*^	0.07 ± 0.3^a^	0.24 ± 0.4^b^
9. Leucine^*∗*^	0.12 ± 0.34^a^	0.40 ± 0.3^b^
10. Tyrosine	0.02 ± 0.25^a^	0.08 ± 0.2^b^
11. Phenylalanine^*∗*^	0.05 ± 0.2^a^	0.18 ± 0.34^b^
12. Histidine^*∗*^	0.18 ± 0.15^a^	0.60 ± 0.2^b^
13. Lysine^*∗*^	0.12 ± 0.4^a^	0.58 ± 0.2^b^
14. Arginine	0.06 ± 0.3^a^	0.66 ± 0.6^b^
15. Total amino acids (%)	1.275 ± 0.24	6.18 ± 0.3
AA essential (%)	0.95 ± 0.27	3.60 ± 0.2
AA nonessential (%)	0.325 ± 0.35	2.58 ± 0.15

^*∗*^amino acids essential. Description: M0 (control), M2(fish meal 8%): the different letters within the same column represent significantly different values (*p* < 0.05).

**Table 6:** The fatty acid content of sago noodles.

Fatty acids	M_0_ (%)	M_2_ (%)
1. Caprylic acid, C8:0	0.061 ± 0.1^a^	0.189 ± 0.3^b^
2. Lauric acid, C12:0	0.008 ± 0.2^a^	0.024 ± 0.4^b^
3. Tridecanoic acid, C13:0	0.00 ± 0.3^a^	0^a^
4. Myristic acid, C14:0	0.15 ± 0.23^a^	0.51 ± 0.12^b^
5. Myristoleic acid, C14:1	0.004 ± 0.4^a^	0.0156 ± 0.1^b^
6. Pentadecanoic acid, C15:0	0.06 ± 0.56^a^	0.27 ± 0.1^b^
7. Palmitic acid, C16:0	0.59 ± 0.2^a^	2.97 ± 0.3^b^
8. Palmitoleic acid, C16:1	0.13 ± 0.14^a^	0.42 ± 0.2^b^
9. Heptadecanoic acid, C17:0	0.07 ± 0.45^a^	0.21 ± 0.25^b^
10. Cis-10-heptadecanoic acid, C17:1	0.03 ± 0.3^a^	0.09 ± 0.3^b^
11. Stearic acid, C18:0	0.50 ± 0.27^a^	1.8 ± 0.6^b^
12. Elaidic acid, C18:1n9t	0^a^	0^a^
13. Oleic acid, C18:1n9c	0.75 ± 0.3^a^	0.46 ± 0.5^b^
14. Linolelaidic acid, C18:2n9t	0.02 ± 0.1^a^	0.06 ± 0.6^b^
15. Linoleic acid, C18:2n6c	0.06 ± 0.5^a^	0.18 ± 0.3^b^
16. Arachidic acid, C20:0	0.02 ± 0.4^a^	0.06 ± 0.2^b^
17. y-linolenic acid, C18:3n6	0.00 ± 0.3^a^	0
18. Cis-11-eicosenoic acid, C20:1	0.04 ± 0.2^a^	0.03 ± 0.1^b^
19. Linolenic acid, C18:3n3	0.011 ± 0.25^a^	0.039 ± 0.4^b^
20. Heneicosanoic acid, C21:0	0.008 ± 0.4^a^	0.024 ± 0.5^b^
21. Cis-11,14-eicosedienoic acid, C20:2	0.022 ± 0.3^a^	0.066 ± 0.34^b^
22. Behenic acid, C22:0	0.025 ± 0.25^a^	0.081 ± 0.1^b^
23. Cis-8,11,14-eiosetrienoic acid, C20:3n6	0^a^	0^a^
24. Cis-11,14,17-eicosatrienoic acid methyl ester, C20:3n3	0^a^	0^a^
25. Arachidonic acid, C20:4n6	0.026 ± 0.1^a^	0.093 ± 0.4^b^
26. Tricosanoic acid, C23:0	0^a^	0^a^
27. Cis-5,8,11,14,17-eicosapentaenoic acid, C20:5n3	0.24 ± 0.1^a^	1.14 ± 0.46^b^
28. Lignoceric acid, C24:0	0.015 ± 0.2^a^	0.045 ± 0.3^b^
29. Nervonic acid, C24:1	0.011 ± 0.1^a^	0.033 ± 0.21^b^
30. Cis-4,7,10,13,16,19-docosahexaenoid acid, C22:6n3	0.41.23 ± 0.25^a^	1.26 ± 0.1^b^

Description: M0 (control), M2(fish meal 8%): the different letters within the same column represent significantly different values (*p* < 0.05).

## Data Availability

On request, the data used to support the findings of this study can be obtained from the corresponding author.
